# Thematic analysis of narrated reasons for suicidal ideation among Chinese adolescents in psychological counseling

**DOI:** 10.3389/fpsyt.2025.1637267

**Published:** 2025-09-29

**Authors:** Li Guo, Nan Zhang, Shuzhen Chen

**Affiliations:** ^1^ School of Foreign Languages, Linyi University, Linyi, Shandong, China; ^2^ Faculty of Language and Linguistics, University Malaya, Kuala Lumpur, Malaysia

**Keywords:** suicidal ideation, Chinese adolescents, thematic analysis, qualitative research, mental health, psychological counseling

## Abstract

**Background:**

Adolescent suicidal ideation is a critical public health concern in China, yet the subjective drivers of these thoughts remain insufficiently understood.

**Objective:**

This study sought to elucidate the reasons Chinese adolescents articulate for suicidal ideation in real‐world counseling contexts.

**Methods:**

We performed a reflexive thematic analysis of therapy transcripts from 51 adolescents (aged 10–19 years; 38 females and 13 males) seeking counseling for depressive symptoms, recruited from the psychiatric outpatient departments of Grade A tertiary hospitals in China.

**Results:**

The analysis identified five interconnected themes: (1) self-cognitive dissonance, (2) academic pressure, (3) family factors, (4) mental health issues (referring to the direct impact of their mental health symptoms), and (5) weak social ability. The findings reveal that these themes operate as a synergistic system, creating a reinforcing cycle where external pressures exacerbate internal vulnerabilities. For these adolescents, suicidal ideation appears to emerge from the complex interplay of academic, familial, social, and intrapsychic distress.

**Conclusions:**

The results provide a nuanced understanding of how multiple risk factors synergize in this population and underscore the necessity of adopting integrated, multi-systemic interventions that concurrently target the adolescent’s cognitions, their family dynamics, and the school environment to effectively address this multifaceted crisis.

## 1 Introduction

### 1.1 The escalating concern of adolescent mental health and suicidal ideation globally and in china

Adolescence is a developmental period of heightened vulnerability for mental health challenges, with suicidal ideation representing a severe manifestation of distress and a key predictor of suicide attempts (1, 2). Globally, an estimated 14% of adolescents experience mental disorders (3), yet the prevalence and expression of this distress vary significantly across cultural landscapes (4). In China, the issue has reached a critical point, with recent meta-analyses reporting a pooled suicidal ideation prevalence of 18.6% among adolescents (5), alongside alarming rates of non-suicidal self-injury (6).

These statistics, while stark, do not fully capture the subjective experience of suffering. To develop effective interventions, it is crucial to move beyond identifying risk factors and toward understanding the lived realities of these adolescents. This is particularly true within China’s unique sociocultural context, where factors such as intense academic competition, traditional family expectations, and cultural concepts of ‘face’ and social harmony uniquely shape both the nature of psychological distress and its articulation (7). This study is grounded in the perspective that adolescent suicidality in this context is best understood not as a result of isolated stressors, but as an outcome of a synergistic system of pressure. Therefore, a deeper exploration of how Chinese adolescents narrate the reasons for their suicidal ideation is essential for uncovering the intricate interplay of these personal and sociocultural forces.

### 1.2 Psychological counseling as a window into subjective experience of adolescent’s suicidal ideation

Psychological counseling and psychotherapy are established as effective interventions for adolescent mental distress and are often a primary treatment choice in China (8, 9). The therapeutic setting provides a unique space for inquiry. It is a co-constructed environment where an adolescent’s narrative of distress is shaped both by their internal experience and by the therapeutic process itself, which encourages the verbalization of thoughts and feelings (10). Analyzing these dialogues offers insight not only into the adolescent’s subjective world but also into how distress is articulated when prompted by a clinician. These conversations are thus an invaluable source of rich, ecologically valid data.

It is within these interactions that adolescents provide first-hand accounts of the subjective reasons and lived experiences they themselves link to their suicidal ideation (11). Analyzing the core patterns and recurrent themes within these subjective narratives is therefore essential for developing interventions that are attuned to the issues adolescents deem most salient. This study leverages the therapeutic dialogue to systematically identify and understand the key thematic content that characterizes the narratives of Chinese adolescents struggling with thoughts of suicide.

### 1.3 Understanding adolescent suicidal narratives: current research and identified gaps

Extensive research has identified a wide array of risk factors for adolescent suicidal ideation across individual, familial, and socio-cultural domains (12, 13). However, much of this knowledge is derived from quantitative studies using questionnaires, which often pre-determine the areas of inquiry and may not capture the full complexity of an adolescent’s subjective experience. While qualitative studies have provided deeper insights into the mental health experiences of Asian youth, including explorations of stigma and self-concept (14, 15), a significant gap remains.

Few studies have systematically analyzed the spontaneously articulated reasons for suicidal ideation as they emerge within the confidential and ecologically valid context of psychotherapy, particularly in China. Understanding the key themes that adolescents themselves prioritize when explaining their profound distress to a therapist is critical for aligning clinical interventions with their perceived realities and needs. This study, therefore, moves beyond pre-defined risk factors to explore the salient, client-generated themes that characterize these crucial conversations. By focusing on “story units”—coherent narrative segments detailing events, thoughts, and feelings (11)—our thematic analysis aims to illuminate the core components of the distress narratives of Chinese adolescents.

### 1.4 The present study: aim and research questions

Given the high incidence of suicidal ideation among Chinese adolescents and the unique insights afforded by therapeutic dialogue, this study was motivated by the need to move beyond quantitative risk-factor identification toward a qualitative understanding of adolescents’ subjective realities. The present study therefore aims to explore and analyze the articulated reasons and associated experiences underlying suicidal ideation as narrated by Chinese adolescents within psychological counseling sessions. Through a systematic thematic analysis focusing on narrative “story units,” this research seeks to illuminate how these young individuals make sense of and communicate their profound distress. Specifically, this study addresses the following research questions:

1. *What are the primary themes that Chinese adolescents articulate when explaining the reasons for their suicidal ideation during psychological counseling?*
2. *How do adolescents describe the* sp*ecific experiences and contexts associated with these primary themes within their narrative “story units”?*

## 2 Methods

### 2.1 Study design

This study employed an inductive qualitative design, utilizing reflexive Thematic Analysis (TA) as the methodological framework (16, 17). This approach was chosen for its flexibility and rigor in identifying, analyzing, and interpreting patterns of meaning (themes) within rich qualitative data. A reflexive TA approach acknowledges the active role of the researcher in knowledge production and is particularly suited for exploring the nuanced subjective experiences of participants (17). For this study, the analysis focused on “story units”—operationally defined for this study as a coherent, multi-turn conversational segment where a participant described a specific past event or a recurring internal state explicitly linked to suicidal ideation. To be coded as a unit, the segment had to contain both a descriptive component (what happened) and an affective component (how it felt).

### 2.2 Data source and participants

The development of the Linyi University Doctor-Patient Conversation Corpus, from which our data were drawn, was approved by the Linyi University Academic Ethics Committee (Approval No: LYU2018001) and has been under development since 2018 to analyze doctor-patient communication. This large-scale corpus project adhered to strict ethical protocols throughout its data collection phase. In line with these protocols, informed consent was obtained from all participants. As the participants in this study are minors, this required a specific two-part process: written informed consent was secured from a legal guardian, and subsequently, written informed assent was obtained directly from the adolescent.

The participating clinicians were experts from the psychiatric outpatient departments of Grade A tertiary hospitals, trained in an integrative modality that primarily draws on principles of Cognitive Behavioral Therapy (CBT) and person-centered therapy. This therapeutic context is a crucial feature of our data. CBT, in particular, actively guides clients to identify and articulate negative automatic thoughts and core beliefs. Therefore, the resulting transcripts are not a record of spontaneous monologue but of a dialogue structured to elicit specific types of cognitive and affective content. Our analysis accounts for this by interpreting themes within the context of this therapeutic elicitation.

For the present study, a purposive sampling strategy was employed to select a relevant subset of transcripts. The inclusion criteria were as follows: (a) the adolescent participant was between 10 and 19 years old; (b) the adolescent was seeking counseling for significant depressive symptoms; and (c) the adolescent explicitly discussed suicidal ideation (e.g., thoughts of dying, wanting to end their life) at least once during the recorded session. The identification of ‘significant depressive symptoms’ was based on the treating clinician’s professional judgment, which was informed by pre-counseling assessments indicating varying degrees of depressive tendencies. It is important to note that while these initial assessments were conducted, the secondary dataset available for this study did not include scores from specific standardized diagnostic interviews (e.g., SCID) or symptom severity scales (e.g., BDI-II). Consequently, our findings should be understood as reflecting the narratives of adolescents seeking therapeutic help for depressive experiences, rather than a formally diagnosed cohort of major depressive disorder.

This process yielded 51 unique consultation transcripts. While we aimed for gender balance during sample selection, the available corpus contained significantly fewer male adolescents seeking therapy. To maintain the scientific validity of our thematic exploration without being skewed by a very small male subgroup, we prioritized the age criterion (10–19 years) and included all available transcripts that met the inclusion criteria, resulting in the current gender distribution. All identifying information was removed or pseudonymized during transcription to ensure complete anonymity. The demographic and clinical characteristics of the sample are summarized in **Table 1**. The pronounced gender imbalance (74.5% female) is a significant feature of this clinical dataset and may reflect gender differences in help-seeking behaviors in China. The potential influence of this gender distribution on the study’s thematic findings is acknowledged as a key limitation and will be discussed in detail.

**Table 1:** Participant characteristics (N = 51).

Characteristic		Value
Age (years)	Range	10–19
Gender, n (%)	Female	38 (74.5%)
	Male	13 (25.5%)
Clinical Presentation, n (%)	Clinician-identified depressive symptoms	51 (100%)
	Explicitly mentioned SI	51 (100%)

### 2.3 Data analysis

The analysis of the 51 transcripts was conducted using the six-phase process for reflexive Thematic Analysis (TA) as detailed by Braun and Clarke (16), facilitated by NVivo 12 software. Prior to analysis, all transcripts were meticulously transcribed and anonymized according to standardized protocols, with cross-verification by multiple team members to ensure accuracy. The data were then stored securely to maintain confidentiality. For this report, all illustrative quotes were translated from Mandarin to English and subsequently validated by a bilingual expert fluent in both languages to ensure the translation accurately conveyed the original meaning and nuance. The six phases were as follows:

1. Familiarization: members of the research team independently read the full dataset to achieve deep immersion, documenting initial thoughts in analytical memos.
2. Systematic Coding: One researcher conducted a comprehensive, line-by-line coding of the entire dataset using an inductive approach.
3. Generating Initial Themes: The research team collaboratively reviewed the codes, sorting and grouping them into potential themes and creating initial thematic maps. To substantiate the interconnected nature of the themes, we systematically coding for the presence of each theme within every identified story unit to map the relationships most frequently articulated by participants.
4. Reviewing and Refining Themes: Potential themes underwent a two-level review to ensure coherence, distinctiveness, and accurate representation of the dataset.
5. Defining and Naming Themes: The team collaboratively wrote detailed definitions and concise names for each finalized theme and subtheme.
6. Producing the Report: The final phase involved writing the narrative analysis, including selecting compelling quotes to illustrate the themes.

In line with the principles of reflexive TA, which values depth and interpretive nuance over consensus, rigor was established through several qualitative strategies (17, 18). The primary strategy was sustained peer debriefing. The full research team (N.Z., S.C., and L.G.) held regular, intensive meetings throughout the analytical process. In these sessions, the primary coder’s interpretations were challenged, alternative explanations were explored, and the thematic structure was collaboratively refined. This iterative process ensured that the final themes were not idiosyncratic but were a robust and thoughtfully considered representation of the data.

The research team approached this analysis from a mixed linguistic and educational psychology perspective. Through sustained peer debriefing, we continuously challenged our interpretations to move beyond surface-level descriptions. For instance, initial codes related to family conflict, such as “parents quarrel” and “parents blame the child,” could have been simply grouped under a general theme of ‘stressful home environment.’ However, our reflexive discussions prompted a deeper analysis. We focused on how adolescents narrated their role within these conflicts—not just as passive observers, but as actors who felt responsible or like a burden. This led to the refinement of the “Family Factors” theme to specifically highlight the adolescent’s experience of being emotionally burdened or blamed, which proved to be a more potent and psychologically significant aspect of their distress. This iterative process of questioning and deepening our understanding ensured a nuanced and rigorously developed analysis.

### 2.4 Ethical considerations

This study was conducted in strict accordance with the ethical principles outlined in the Declaration of Helsinki and received full prior approval from the Linyi University Academic Ethics Committee (Approval No: LYU2018001; Dated: December 30, 2018).

Given the vulnerability of the adolescent participants, a rigorous two-part consent process was implemented during the original data collection. First, written informed consent was obtained from a legal guardian for every participant. Second, written informed assent was obtained directly from each adolescent participant. This process involved a comprehensive explanation of the study’s purpose, the voluntary nature of participation, and their right to withdraw at any time without penalty. It was explicitly stated that their decision would not impact their clinical care. To ensure confidentiality, all personal identifiers were removed from transcripts and replaced with alphanumeric codes.

## 3 Results

**Figure 1:**
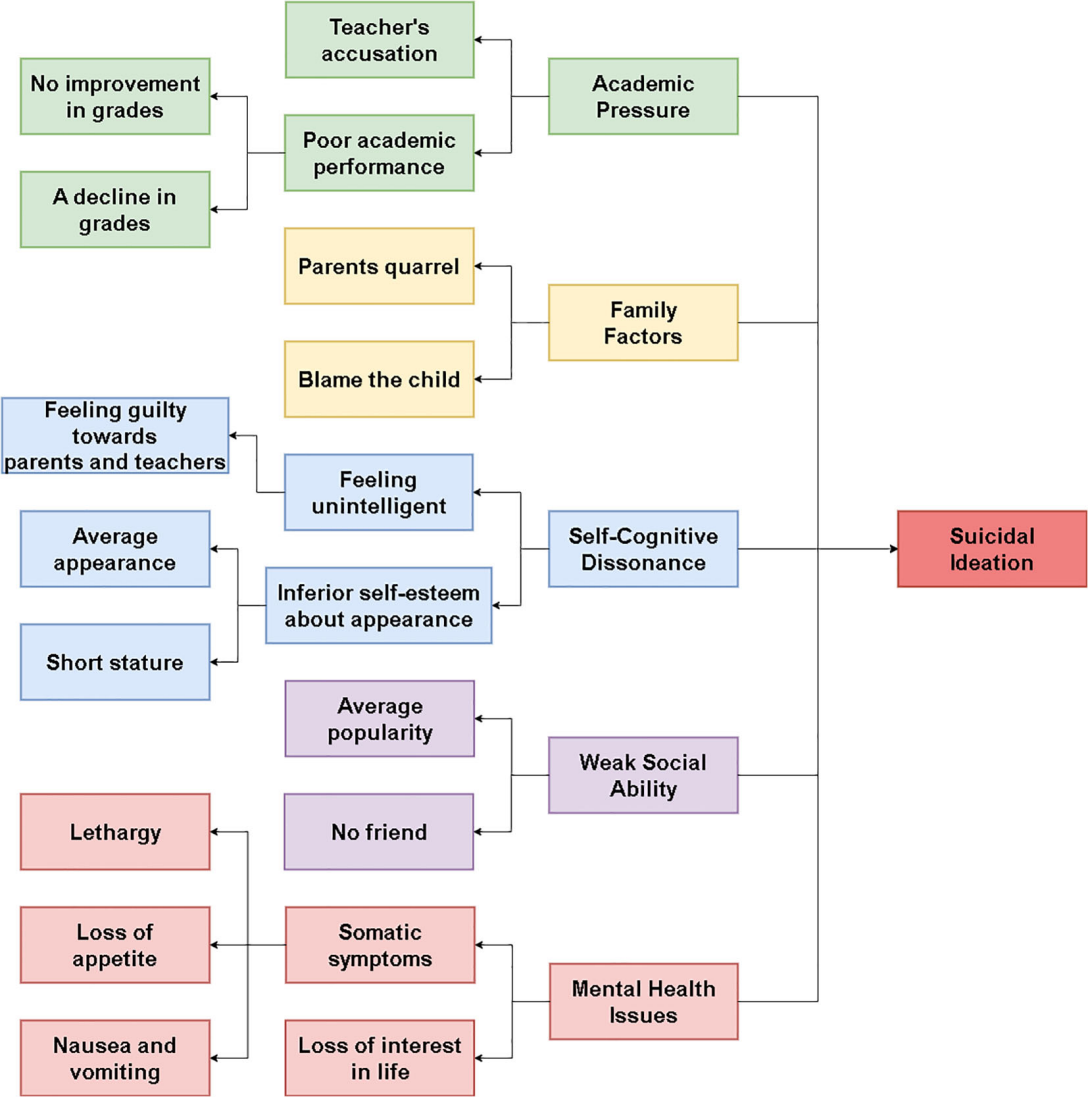
Thematic map of the synergistic system of distress leading to suicidal ideation.

The thematic analysis of the 51 counseling transcripts identified five primary themes representing the articulated reasons and experiences that Chinese adolescents associated with their suicidal ideation. These themes are: (1) self-cognitive dissonance, (2) academic pressure, (3) family factors, (4) mental health issues, and (5) weak social ability. While these themes are presented separately for analytical clarity, the adolescents’ narratives consistently revealed their deep interconnectedness. Due to space constraints, one illustrative example will be presented in detail for each primary theme to elucidate its core characteristics, while other related subthemes (as depicted in **Figure 1**) will be briefly contextualized. Participant identifiers (e.g., P=patient, D=doctor) are used to ensure anonymity.

### 3.1 Self-cognitive dissonance

Self-cognitive dissonance emerged as a significant theme, reflecting adolescents’ internal struggles with a profoundly negative self-perception. This was often characterized by a perceived lack of worth, abilities, or positive attributes, leading to pervasive feelings of inadequacy and hopelessness, concepts strongly linked to increased vulnerability to suicidal ideation (19–23). This theme encompassed feeling unintelligent or harboring inferior self-esteem, particularly concerning appearance (which, as indicated in **Figure 1**, could relate to aspects such as perceived short stature or average appearance), and often resulted in feelings of guilt or being a disappointment.

A stark illustration of this profound lack of positive self-regard and its verbal manifestation was evident in an exchange between a doctor (D) and a female adolescent (P):

D: Oh, not interested in anything. How do you evaluate yourself? (1.4) Hmm, how do you evaluate yourself? (0.6) What do you think your advantages are compared to your deskmates around you?
P: No advantages.
D: No advantages, huh.
P: Yes.
D: Let me say something, (1.2) see if it’s true. Do you look younger than them?
P: No.
D: No, huh. Do you seem to study better than them?
P: No.
D: Not that either, huh. Mm. Do you seem more sensible than them here?
P: No.
D: Not that either. Mm. So, you feel you are not as good as them in any aspect?
P: No.
D: Not that either, hehehe. Think about where your advantages lie? (3.5) Do you have any merits?
P: No.

(See **Appendix** for the original Mandarin transcript).

The dialogue starkly demonstrates the totality of this adolescent’s negative self-concept. Her responses are not hesitant or elaborate; they are brief, categorical negations (“No,” “No advantages”) that function as a rhetorical fortress, systematically rejecting the therapist’s attempts to co-construct a more positive identity. The final, stark “No,” underscores the absolute nature of her self-assessment. This actively defended position of global worthlessness serves as a powerful illustration of the cognitive distortions central to depression. It directly exemplifies the ‘negative view of self’ within Beck’s cognitive triad and is strongly linked to the profound hopelessness identified as a key proximal risk factor for suicide (24, 25). The analysis reveals how such an all-encompassing negative self-view, impervious to external validation, forms a powerful cognitive basis for the belief that one’s life is without value, thereby directly contributing to suicidal ideation.

### 3.2 Academic pressure

Academic pressure was a pervasively cited source of distress contributing to suicidal ideation among the adolescents in this study. This theme encompassed intense stress related to academic performance, examinations, and a perceived failure to meet the high expectations often placed upon students in competitive educational systems (26). While subthemes included the direct impact of poor academic performance (manifesting as no improvement or a decline in grades, as shown in **Figure 1**), a particularly impactful aspect highlighted by participants was the experience of negative interactions with educators, such as Teacher’s Accusation. Such interactions can be especially damaging to adolescents’ self-esteem and psychological well-being (27, 28).

One participant (female, former high school entrance exam candidate) powerfully recounted how a teacher’s criticism during a crucial examination period directly fueled her distress:

P: Then I took the exam, and then I took the high school entrance exam. Actually, the period of time when I took that exam was (0.5) the most depressing. It wasn’t me who was depressed. It was a teacher. I took tutoring classes with him. Then (0.5) my grades didn’t seem to work. That is (0.5) my grades didn’t drop or improve, they didn’t change. Maybe he was not satisfied, so he kept criticizing me, and then he talked about me in front of many people (0.2). He said, “Why are you still attending tutoring classes? Stop it. You’re just wasting your mother’s money”.

(See **Appendix** for the original Mandarin transcript).

This narrative vividly illustrates how academic struggles can be transformed into a profound personal failing through public criticism from a significant authority figure. The teacher’s reported speech is particularly damaging. The rhetorical question, “Why are you still attending tutoring classes?” serves to invalidate her efforts, while the final statement, “You’re just wasting your mother’s money,” reframes her as both a financial and a moral burden.

This experience aligns with research showing that perceived invalidation and public shaming from educators can severely undermine an adolescent’s self-esteem and foster a sense of academic helplessness, directly precipitating suicidal thoughts (29, 30). The cultural context intensifies this damage. The specific phrasing “You’re just wasting your mother’s money,”, carries a particularly sharp weight. The term ‘waste money’ implies being a drain, a useless expenditure. In a cultural context where filial piety (xiào) and contributing to family honor are paramount, being framed as a net drain on one’s mother is a profound accusation of filial failure, striking at the core of the adolescent’s social and familial identity.

### 3.3 Family factors

Family-related issues, particularly stressful home environments and negative parental dynamics, were frequently implicated in adolescents’ narratives of distress. While this theme broadly encompassed issues like overt parental conflict, a particularly powerful subtheme was the adolescent’s experience of being emotionally burdened or blamed within these conflicts. Rather than being a source of stability, the family environment was often described as a space of emotional turmoil and divided loyalties, contributing significantly to feelings of insecurity and psychological distress that fueled suicidal thoughts (31–33), as illustrated by one participant’s account of feeling pressured to take sides after a parental quarrel:

P: I think (0.5) um, maybe (0.2) I think it might not be caused by me, but deep down I might feel that there is a little related, because I always remember that my mother had a quarrel with my father a long time ago, and after the quarrel, she saw me and asked me why I didn’t go to support her.

(See **Appendix** for the original Mandarin transcript).

This excerpt illustrates how the adolescent internalizing parental conflict. The participant’s opening, marked by hesitation and self-correction (“I think (0.5) um, maybe…”), signals his cognitive struggle to assign blame. He consciously attempts to absolve himself (‘it might not be caused by me’), yet immediately reveals a deeper, contradictory feeling: “but deep down I might feel that there is a little related.” He links this irrational yet potent sense of responsibility to a specific memory: his mother confronting him for not taking her side. This places the adolescent in an untenable position of divided loyalties, implicitly blaming him for his lack of “support”.

Such family dynamics, where an adolescent is drawn into parental disputes and feels an undue sense of responsibility, are known to foster profound emotional insecurity and instability (34). The guilt and emotional burden from being unable to satisfy parental expectations in these conflicts can become overwhelming. In this context, the adolescent is recast not merely as a bystander, but as a participant who has failed in his filial duty, making the home environment feel emotionally unsafe and contributing to suicidal ideation as a perceived escape (33).

### 3.4 Mental health issues

Beyond external stressors, a distinct theme emerged where adolescents attributed their suicidal ideation directly to the relentless and overwhelming nature of their internal mental health symptoms. Their narratives described how the lived, moment-to-moment experience of their condition—including loss of interest in life, and debilitating somatic symptoms like lethargy and loss of appetite—made continued existence feel unbearable (35). In these accounts, suicide was framed not as a response to a specific problem, but as an escape from a painful and exhausting internal state itself.

The following exchange between a doctor and a patient illustrates how the persistent and cyclical nature of low mood and associated symptoms like loss of appetite contributed to suicidal feelings:

D: At that time, why did you think about suicide?
P: I just felt so tired.
D: Can you recover from this tiredness after sleeping?
P: In the morning, if I have to go to school, my mood might be quite good the night before, but when I wake up in the morning, my mood is very low.
D: Oh, low in the morning. What about in the evening?
P: It’s quite good in the evening.
D: Quite good in the evening. Have you eaten?
P: Anyway, I haven’t had much appetite recently.

(See **Appendix** for the original Mandarin transcript).

The participant’s initial explanation for his suicidal thoughts is profoundly simple yet powerful: “I just felt so tired.” This is not a description of normal fatigue but a state of deep, existential exhaustion that makes continued existence feel unbearable. His subsequent description of classic depressive symptoms—specifically, a pronounced diurnal mood variation (feeling low in the morning) and loss of appetite—paints a picture of a body and mind under siege from within. This lived experience of persistent, oppressive internal discomfort aligns with clinical research highlighting how the direct, debilitating impact of depressive symptoms can itself become a primary driver for suicidal ideation (36, 37). His narrative constructs a reality where even the basic restorative functions of life, like sleep and food, offer no respite or pleasure. For this adolescent, the desire to die stems directly from the sheer, unending burden of the condition itself.

### 3.5 Weak social ability

The final theme captured adolescents’ profound struggles with social interactions and the formation of meaningful peer relationships. This went beyond typical teenage feelings of unpopularity; it was characterized by a deep-seated sense of social inadequacy and isolation. Subthemes included experiences of having no friends and feeling of average popularity (implying a lack of significant connection). For some participants, this perceived deficit in social functioning was not just a contributing factor but was identified as the primary, originating cause of their depression and subsequent suicidal ideation, representing a core failure in a critical domain of adolescent life (38).

One participant directly linked his depression and suicidal feelings to an inability to cope with social environments:

P: (1.0) Ah, then then, let’s count it as my social problems. I think, I think what, my depression, my depression, to be precise, comes from social interaction. I really, I really can’t get used to these people, that’s why I’m depressed. Then what my mom told me was for me to get used to those people, and then after I got used to them for several years, I was still depressed. From my first year of junior high until now, it’s been several years, and I’m still depressed.

(See **Appendix** for the original Mandarin transcript).

This participant’s narrative functions as a powerful causal argument. He opens by explicitly labeling his issue as “my social problems,” and through repetition and self-correction, culminates in a definitive etiological statement: “my depression … comes from social interaction.” Crucially, his narrative also highlights the failure of simplistic solutions. His mother’s advice to “get used to” others is framed as a long-term experiment that has definitively failed, reinforcing his sense of hopelessness.

This experience powerfully illustrates how a perceived, intractable failure in the social domain can become the central organizing narrative for an adolescent’s entire experience of suffering (39). The final statement, emphasizing the multi-year duration of his struggle, serves to construct his condition as chronic and unchangeable. By framing the problem as both fundamental to his being and unsolvable by conventional means, his narrative makes suicidal thoughts appear as a rational escape from what feels like an inescapable reality of social inadequacy (40).

### 3.6 Interconnectedness of themes

While the five themes are presented separately for analytical clarity, they rarely appeared in isolation within the adolescents’ narratives. It was common for participants to articulate multiple, overlapping reasons for their suicidal ideation, suggesting that these factors often operate in tandem. For instance, sustained academic pressure could exacerbate underlying mental health issues or contribute to negative self-cognitions (41). Similarly, family conflicts might lead to social withdrawal and a diminished sense of self-worth (42–44). This observation aligns with existing research, which highlights that the accumulation and interplay of various risk factors often intensify an adolescent’s suffering and subsequent suicidal ideation (45). This dynamic suggests that the capacity to cope with stress may function as a limited resource; when multiple stressors compete for this resource, it becomes depleted, leading to the adolescents feeling overwhelmed and helpless. This multifaceted nature of their distress underscores the complexity of addressing adolescent suicidality. Further investigation into how these themes dynamically interact would be a valuable next step in understanding this complex issue.

## 4 Discussion

These findings offer a nuanced, in-depth perspective on the subjective experiences of distress that Chinese adolescents associate with their thoughts of suicide, providing critical insights that complement and expand upon existing quantitative and broader qualitative research.

This thematic analysis of therapeutic narratives provided critical insight into the subjective experiences underlying suicidal ideation among Chinese adolescents. The analysis yielded five prominent and interconnected themes: self-cognitive dissonance, academic pressure, family factors, mental health issues, and weak social ability. In the following discussion, we will first situate these five themes within the existing literature, highlighting how our findings both corroborate and culturally specify established theories of suicidality. We will then reflect on the methodological significance of analyzing therapeutic narratives, before exploring the profound implications of our findings for clinical practice, prevention policy, and future research.

### 4.1 Echoes and elaborations: situating findings within existing literature

The current findings both corroborate and extend existing literature on adolescent suicidal ideation through a culturally situated lens. Five key themes emerged from participants’ narrative constructions, each demonstrating important connections to established theoretical frameworks while revealing culture-specific manifestations.

First, the theme of Self-Cognitive Dissonance substantiates cognitive theories of suicide (24, 25), with participants exhibiting profound negative self-schemas characterized by pervasive self-devaluation. However, our findings add cultural texture. The absolute and global nature of the self-devaluation seen in our data suggests that in a high-achievement, collectivistic society, the cognitive distortions associated with depression may be particularly severe. Analysis of story units revealed this as an active process of self-invalidation resistant to therapeutic intervention, highlighting the entrenched nature of these maladaptive cognitions (20, 21).

Second, Academic Pressure emerged as particularly salient within China’s competitive educational context (46). Our analysis revealed that the damage extended beyond general academic stress (47). Specific, narrated interactions, such as public shaming by a teacher, transformed academic struggles into profound threats to an adolescent’s social standing and “face” (48)—a concern of paramount cultural significance (7).

Third, the theme of Family Factors reveals a critical paradox, challenging simplistic assumptions about the universally protective role of the family. While family systems are often positioned as a primary source of support (31), our participants’ narratives illustrated how they can transform into a crucible of distress. Experiences of interparental conflict and emotional burdening were particularly potent, reframing the home from a safe haven into an aversive environment characterized by divided loyalties (32, 33). This finding underscores that for these adolescents, the family did not buffer against stress but instead became a direct and significant contributor to it.

Fourth, Mental-Health Issues narratives emphasized the phenomenological experience of depression (36), in these accounts, suicidal ideation was not framed as a response to a specific life problem, but as a direct and desperate escape from the suffocating internal state of the illness itself (37).

Finally, the theme of Weak Social Ability aligned with established research on the critical role of peer relationships in adolescent mental health (49). Participants’ narratives of profound loneliness and an inability to form meaningful connections highlighted a core developmental vulnerability during this sensitive, identity-forming period (1). For these adolescents, the perceived failure in the social domain was not just a source of sadness, but was often constructed as a fundamental flaw in their character, leading to intense feelings of isolation and alienation that fueled their suicidal ideation.

Critically, this study’s primary contribution lies in revealing how these five themes do not operate in isolation, but as a synergistic system. The narratives consistently showed these factors creating a cascading effect that amplifies feelings of hopelessness and entrapment, a key mechanism in suicidal desire (45, 50). This profound interconnectedness underscores that effective interventions cannot target these issues individually, but must adopt a multi-systemic approach that simultaneously addresses the adolescent’s internal world, family dynamics, and school environment.

### 4.2 The significance of articulated narratives in counseling

Our methodological approach, centered on analyzing “story units” from within therapy, carries significant clinical implications. While quantitative surveys can identify risk factors, a thematic analysis of spontaneously generated narratives provides an ecologically valid window into the subjective world of a suicidal adolescent. The themes identified in this study are not merely researcher-imposed constructs; they represent the causal explanations and salient issues that adolescents themselves prioritize when trying to make sense of their suffering (51).

Understanding these client-generated etiologies is foundational to effective clinical work. It allows therapists to build a stronger therapeutic alliance by engaging with the problems the adolescent deems most critical, rather than imposing an external agenda. The therapeutic space is thus a privileged site for both the assessment of these core distress narratives and, crucially, for the potential co-construction of new, more adaptive ones. By listening closely to how an adolescent weaves together themes of academic failure, familial burden, and social isolation, a therapist can begin to help them identify points of intervention within that narrative, challenge hopelessness, and collaboratively author a future story where life is once again perceived as worth living.

### 4.3 Implications for clinical practice, prevention, and policy

The findings from this study, particularly the interconnected nature of the themes, offer a clear map for multi-systemic intervention. At the clinical level, clinicians can move beyond generic support by tailoring specific, evidence-based strategies to address the distinct drivers of distress identified in adolescents’ narratives. For instance, to counter Self-Cognitive Dissonance, therapists can utilize cognitive restructuring techniques from Cognitive Behavioral Therapy (CBT) (52). To mitigate Academic Pressure, psychoeducational interventions can equip adolescents with stress management and self-compassion skills. For distress from Family Factors, systemic approaches like Attachment-Based Family Therapy (ABFT) can restructure family interactions (53). Furthermore, to address the direct burden of Mental Health Issues, psychoeducation and behavioral activation can help normalize the experience and combat lethargy (52). Finally, to address Weak Social Ability, therapists can facilitate structured social skills training, such as the model used in Interpersonal Psychotherapy for Adolescents (IPT-A) (54).

Beyond these individual-level interventions, our findings issue a clear mandate for broader preventive action within the adolescent’s key environments: school and home. Given that narratives frequently cited teachers and parents as direct sources of distress, interventions must extend to these key adults. At the school level, this requires integrating mental health literacy into teacher training and adopting structural reforms that promote diverse definitions of success (55). In parallel, for families, promoting accessible parenting education is essential to equip parents with strategies for constructive communication and conflict resolution, helping them foster a secure home environment (7).

Ultimately, at a broader public health policy level, there is an urgent need to expand accessible youth mental health services across China (8). Policy should also support public campaigns aimed at destigmatizing help-seeking and reinforcing the importance of supportive parenting and educational environments, thereby creating a more holistic ecosystem of care for adolescents.

### 4.4 Strengths, limitations and future directions

This study’s strengths include its use of rich, ecologically valid data drawn from actual psychotherapy sessions, providing deep, client-centered insights into a hard-to-reach population. The rigorous application of thematic analysis, validated through sustained peer debriefing and researcher reflexivity, also ensures the credibility of the findings. However, the study’s limitations must be acknowledged to contextualize the results appropriately.

First, a primary limitation is the pronounced gender imbalance of the sample (74.5% female). This distribution likely reflects known gender differences in help-seeking, as adolescent females in China are more likely to access clinical services (8). This is notable given that national mortality data show an increasing male-to-female ratio of suicide deaths (56). Consequently, our findings may not be fully generalizable to male adolescents, whose experiences are underrepresented. The synergistic system we describe must therefore be understood as potentially skewed toward a female-typical presentation of suicidality.

A second limitation pertains to the therapeutic modality. As noted in the methods, the clinicians utilized an integrative approach primarily based on Cognitive Behavioral Therapy (CBT). This modality actively encourages clients to identify and articulate cognitive distortions, negative automatic thoughts, and core beliefs. Consequently, the prominence of themes such as “Self-Cognitive Dissonance” may be, in part, an artifact of the therapeutic process itself, which guided participants to focus on cognitive aspects of their distress. Narratives elicited through other therapeutic approaches (e.g., psychodynamic or humanistic) might have yielded different thematic emphases.

Third, the sample was drawn exclusively from a clinical, help-seeking population. The narratives analyzed are from adolescents who had already accessed mental health services. Their experiences and articulated reasons for suicidal ideation may differ significantly from those of adolescents with similar thoughts who do not or cannot seek professional help due to barriers such as stigma, lack of access, or low mental health literacy. Therefore, the findings may not be representative of the broader population of adolescents experiencing suicidal ideation.

These limitations highlight several key directions for future research. First, to address sample limitations, future studies should employ targeted sampling to achieve a more balanced gender representation and include community-based samples to capture the experiences of non-help-seeking youth. Second, future research would benefit from mixed-methods designs; incorporating validated diagnostic interviews (e.g., the SCID) and symptom severity scales (e.g., the BDI-II) would allow for a quantitative characterization of the sample, complementing the qualitative depth. Finally, longitudinal studies are needed to track how these distress narratives evolve over time, and cross-cultural replications, particularly in low- and middle-income countries (LMICs), would be invaluable for distinguishing universal from culturally-specific drivers of suicidal ideation.

## 5 Conclusion

This thematic analysis of therapeutic narratives provided critical insight into the subjective experiences underlying suicidal ideation among Chinese adolescents. In response to our first research question, the study identified five central themes: self-cognitive dissonance, academic pressures, family factors, mental health issues, and weak social ability. In addressing our second research question, the analysis revealed how adolescents weave these themes together within their “story units” to construct a reality of inescapable suffering.

A key finding that emerged from the analysis is that these themes rarely act in isolation. Instead, the adolescents’ narratives suggest they often form a synergistic system of distress, where external sociocultural pressures—from family and school—amplify internal vulnerabilities, creating a reinforcing cycle of despair. This observation underscores the necessity of moving beyond single-factor explanations and adopting an integrated, ecological perspective in both research and intervention. Ultimately, listening to these narratives teaches us that to prevent suicide, we must not only help adolescents rewrite their personal stories of worthlessness and despair but also strive to change the familial, educational, and societal scripts that place such an unbearable burden upon them.

## Data Availability

The raw data supporting the conclusions of this article will be made available by the authors, without undue reservation.
