# Supplementary material for: Thematic analysis of narrated reasons for suicidal ideation among Chinese adolescents in psychological counseling
Source: Front Psychiatry. 2025 Sep 29;16:1637267. doi: 10.3389/fpsyt.2025.1637267 (PMC12517223; doi:10.3389/fpsyt.2025.1637267)
Supplement: Supplementary file 1 [file SupplementaryFile1.docx]

#### Appendix Original Mandarin Transcripts of Illustrative Quotes

This appendix contains the original Mandarin Chinese transcripts for the illustrative quotes presented in the Results section. The quotes are numbered and correspond to the order in which they appear in the text.

Quote 1 (from Section 3.1 Self-Cognitive Dissonance)

D: 哦，对什么都没兴趣。你怎么评价你自己？ (1.4) 嗯，怎么评价自己？ (0.6) 你觉得你和你前后位几个同桌哈，你比他们的优势在哪里？
P: 没有优势。
D: 没有优势啊。
P: 嗯。
D: 我说一下哈。(1.2) 你看是不是哈。你看起来比他们要年轻？
P: 不是。
D: 不是啊。你看起来学习比他们要好。
P: 不是。
D: 也不是哈。嗯。你看这里比他们更懂事儿。
P: 不是。
D: 也不是。嗯。你看起来什么都不如他们。
P: 不是。
D: 也不是，hehehe。想一想你的优势在哪里？ (3.5) 你有什么优点吗？
P: 没有。

D: Oh, not interested in anything. How do you evaluate yourself? (1.4) Hmm, how do you evaluate yourself? (0.6) What do you think your advantages are compared to your deskmates around you?
P: No advantages.
D: No advantages, huh.
P: Yes.
D: Let me say something, (1.2) see if it’s true. Do you look younger than them?
P: No.

D: No, huh. Do you seem to study better than them?
P: No.
D: Not that either, huh. Mm. Do you seem more sensible than them here?
P: No.
D: Not that either. Mm. So, you feel you are not as good as them in any aspect?
P: No.
D: Not that either, hehehe. Think about where your advantages lie? (3.5) Do you have any merits?
P: No.

Quote 2 (from Section 3.2 Academic Pressure)

P: 然后就考，然后就考高中了，然后就其实考高中那段时间是(0.5)是最压抑了，就是其实不是我压抑，是有一个老师，我在他那里上了辅导班，然后（0.5）就是成绩可能不见效，就是没有掉也没有上去，一直在那，他可能是不满意，然后就老是说我，然后就就在很多人面前说我(0.2)就说你还上什么，你别上了，你就光费你妈的钱。

P: Then I took the exam, and then I took the high school entrance exam. Actually, the period of time when I took that exam was (0.5) the most depressing. It wasn’t me who was depressed. It was a teacher. I took tutoring classes with him. Then (0.5) my grades didn’t seem to work. That is (0.5) my grades didn’t drop or improve, they didn’t change. Maybe he was not satisfied, so he kept criticizing me, and then he talked about me in front of many people (0.2). He said, “Why are you still attending tutoring classes? Stop it. You’re just wasting your mother’s money.”

Quote 3 (from Section 3.3 Family Factors)

P：我觉得（0.5）嗯，可能（0.2）我觉得可能不是我引起的，但是我就心底里可能会觉得有一点关系，因为我一直记得我妈很久以前她跟我爸吵了一次架，然后吵完了以后，然后她看到我，然后她就询问我说不去给她帮腔。

P: I think (0.5) um, maybe (0.2) I think it might not be caused by me, but deep down I might feel that there is a little related, because I always remember that my mother had a quarrel with my father a long time ago, and after the quarrel, she saw me and asked me why I didn’t go to support her.

Quote 4 (from Section 3.4 Mental Health Issues)

D: 那个时候怎么想过自杀？
P: 就感觉好累。
D: 你这个累睡完觉以后能够歇过来吗。
P: 我就早晨如果说我上学的话，我可能晚上心情还挺好的，结果早晨醒来的时候心情就很低落了，
D: 奥，早晨醒来低落，到傍晚呢。
P: 到傍晚就挺好的了
D: 到傍晚就挺好的。吃饭了没有？
P: 反正最近没有什么食欲。

D: At that time, why did you think about suicide?

P: I just felt so tired.

D: Can you recover from this tiredness after sleeping?

P: In the morning, if I have to go to school, my mood might be quite good the night before, but when I wake up in the morning, my mood is very low.

D: Oh, low in the morning. What about in the evening?

P: It’s quite good in the evening.

D: Quite good in the evening. Have you eaten?

P: Anyway, I haven’t had much appetite recently.

Quote 5 (from Section 3.5 Weak Social Ability)

P:（1.0）啊啊，然后然后就算我社交问题了，我认为我认为什么，我抑郁我抑郁准确的说是来自于社交，我实在是我实在是习惯不了这些人，所以我才抑郁。然后我妈告诉我的就是我去习惯那些人，然后我都习惯几年后我还抑郁，我从我的初一到现在我都几年了，我还抑郁。

P: (1.0) Ah, then then, let’s count it as my social problems. I think, I think what, my depression, my depression, to be precise, comes from social interaction. I really, I really can’t get used to these people, that’s why I’m depressed. Then what my mom told me was for me to get used to those people, and then after I got used to them for several years, I was still depressed. From my first year of junior high until now, it’s been several years, and I’m still depressed.
